# Ectopic expression of a grape nitrate transporter VvNPF6.5 improves nitrate content and nitrogen use efficiency in Arabidopsis

**DOI:** 10.1186/s12870-020-02766-w

**Published:** 2020-12-07

**Authors:** Yani He, Xiaojun Xi, Qian Zha, Yuting Lu, Aili Jiang

**Affiliations:** 1 Research Institute of Forestry and Pomology, Shanghai Key Lab of Protected Horticultural Technology, Shanghai Academy of Agricultural Sciences, Shanghai, China; 2 Institute of Plant Physiology and Ecology, Shanghai Institutes for Biological Sciences, Chinese Academy of Sciences, Shanghai, China

**Keywords:** Nitrate, Transporter, Grape, Nitrogen use efficiency, Nitrate signaling

## Abstract

**Background:**

Nitrate plays an important role in grapevines vegetative and reproductive development. However, how grapevines uptake, translocate and utilize nitrate and the molecular mechanism still remains to be investigated.

**Results:**

In this study, we report the functional characterization of *VvNPF6.5*, a member of nitrate transporter 1/peptide transporter family (NRT1/PTR/NPF) in *Vitis vinifera*. Subcellular localization in Arabidopsis protoplasts indicated that VvNPF6.5 is plasma membrane localized. Quantitative RT-PCR analysis indicated that *VvNPF6.5* is expressed predominantly in roots and stems and its expression is rapidly induced by nitrate. Functional characterization using cRNA-injected *Xenopus laevis* oocytes showed that VvNPF6.5 uptake nitrate in a pH dependent way and function as a dual-affinity nitrate transporter involved in both high- and low-affinity nitrate uptake. Further ectopic expression of *VvNPF6.5* in Arabidopsis resulted in more ^15^NO_3_^−^ accumulation in shoots and roots and significantly improved nitrogen use efficiency (NUE). Moreover, VvNPF6.5 might participate in the nitrate signaling by positively regulating the expression of primary nitrate response genes.

**Conclusion:**

Our results suggested that *VvNPF6.5* encodes a pH-dependent, dual-affinity nitrate transporter. VvNPF6.5 regulates nitrate uptake and allocation in grapevines and is involved in primary nitrate response.

**Supplementary Information:**

The online version contains supplementary material available at 10.1186/s12870-020-02766-w.

## Background

Grapevine is grown extensively throughout the world. In 2018, worldwide vineyard area and grape berry production were approximately 7 million hectares and 75 million tons, respectively [1]. As one of the most important nutrients for grapevines, nitrogen has a great impact on vine vegetative and reproductive development as well as grape composition [2, 3]. Sufficient nitrogen stimulates the vigor of the grapevines, which can increase leaf area and leaf chlorophyll, promote photosynthesis and transpiration, and also can delay leaf senescence [4, 5]. Nonetheless, nitrogen over-fertilized grapevines tend to decrease secondary metabolite synthesis, since grapevines become excessively vegetative, increasing the competition between vegetative and reproductive sinks [6]. Moreover, excessive nitrogen fertilizer could result in waste of resources and environmental contamination, and also presents serious hazards for human health [7]. Nitrogen use is complex in grapevines, as each step, including nitrogen uptake, translocation, assimilation, and remobilization, is governed by multiple interacting genetic and environmental factors [7]. To utilize nitrogen efficiently and regulate grapevines development precisely, the mechanisms of nitrogen uptake, allocation and utilization are needed to be well documented.

Nitrate is the major source of nitrogen for most terrestrial plants, especially those grown in aerobic soil conditions [8]. Nitrate concentrations in soil solutions range from very low levels of a few hundred micromolar to around 20 mM, even up to 70 mM [9]. To cope with this wide range of concentrations, plants have evolved two nitrate uptake systems, the high affinity transport system (HATS) and the low-affinity transport system (LATS) [10]. During the past two decades, at least four gene families including nitrate transporter 1/peptide transporter family (NRT1/PTR/NPF), nitrate transporter 2 family (NRT2), chloride channel family (CLC) and slow anion channel-associated homologues (SLAC/SLAH), have been identified to play roles in nitrate transport in higher plants [11].

CHL1 (also named NRT1.1 or NPF6.3) from Arabidopsis was the first identified NRT1/PTR/NPF transporter [12]. As the best-known nitrate transporter, CHL1 displays several unique functional properties. CHL1 could function as a dual-affinity transporter involved in both high- and low-affinity uptake, which was switched by the phosphorylation of T101 [12–14]. Meanwhile, the nitrate sensing function of CHL1 governs the expression of NO_3_^−^-responsive genes and NO_3_^−^-induced changes in root development [15–17]. Moreover, CHL1 could act as an auxin influx facilitator, modulating auxin gradients in lateral root primordia in response to nitrate [18].

Besides CHL1, a number of nitrate transporters have also been reported, which function in different organs or tissues to achieve fine control of nitrate absorption, allocation and utilization. The low affinity nitrate transporters AtNRT1.2, OsNRT1.1 and OsNRT1.1B, and the high affinity nitrate transporters AtNRT2.1, AtNRT2.2, AtNRT2.4, AtNRT2.5, OsNRT2.1, OsNRT2.2 and TaNRT2.1 were all shown to be involved in root nitrate uptake [10]. After being absorbed from soil, nitrate could be subsequent transported to shoots by transporters such as AtNRT1.5, AtNRT1.8, AtNRT1.7, AtNRT1.9, OsNPF2.2, OsNRT2.3a, and LeNRT2.3 [10]. Furthermore, nitrate could be stored in or released from vacuoles by CLCa, CLCb, NRT2.7, NPF5.11, NPF5.12 and NPF5.16 [19]. However, little is known about the nitrate transporters in grapevines.

In this study, *CHL1* homologous gene *VvNPF6.5* in *Vitis vinifera* was identified and functional characterized. Our data revealed that plasma membrane localized VvNPF6.5 was a pH-dependent and dual-affinity nitrate transporter. When ectopic expressed in Arabidopsis, VvNPF6.5 could increase the nitrate content in plants and improve nitrogen use efficiency (NUE). In addition, VvNPF6.5 was proved to be involved in the primary nitrate response.

## Results

### Cloning and sequence analysis of *VvNPF6.5*

**Fig. 1:**
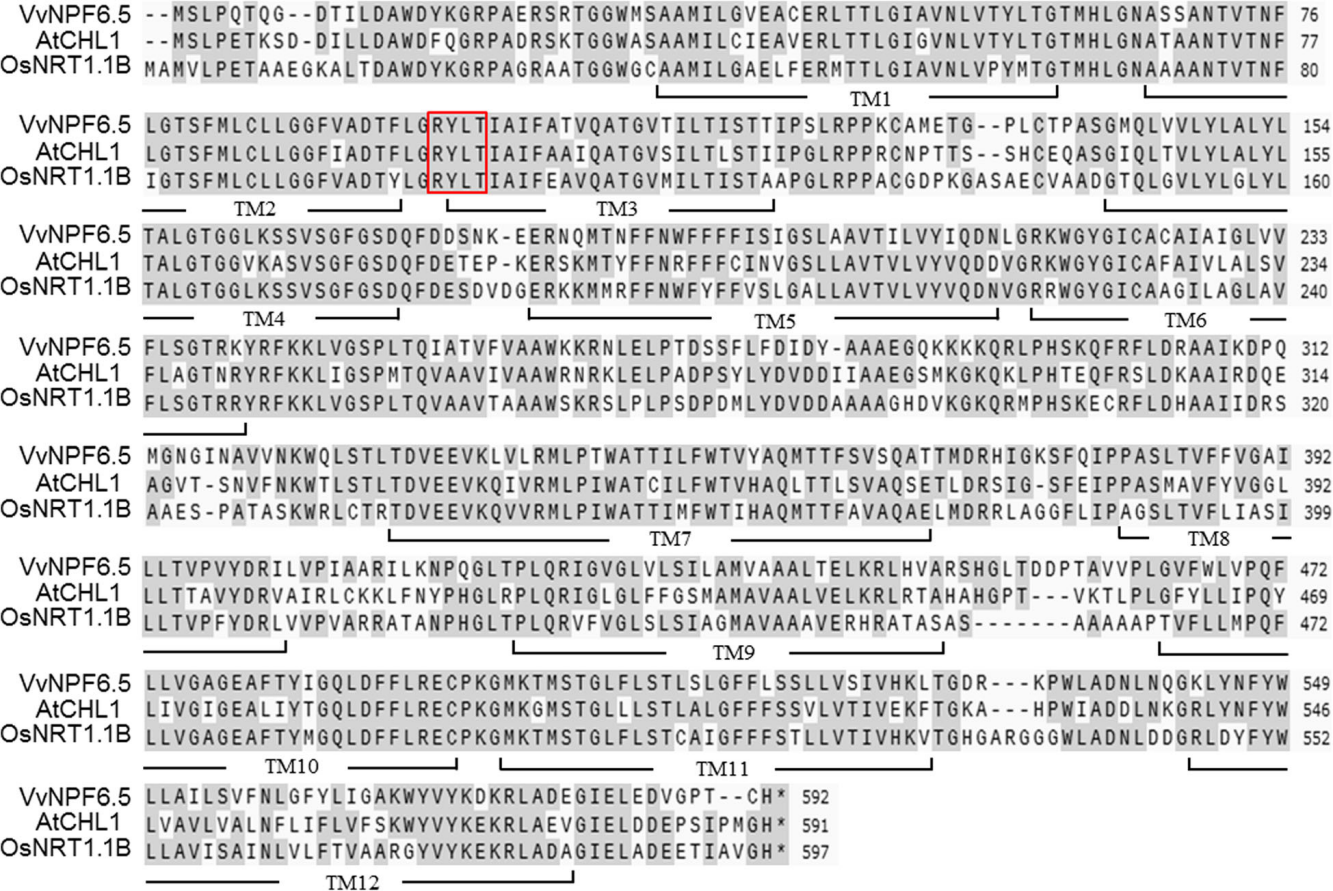
Amino acid sequence alignment of the VvNPF6.5, AtCHL1 and OsNRT1.1B. SnapGene program was used to perform the sequence alignment. The identical residues were shaded and the dots were inserted to optimize the alignment. The putative transmembrane domains (TM) are marked with black underline and are numbered. The putative phosphorylation sites are enclosed in the red box

To explore how grapevines uptake and translocate nitrate, we identified a highly homologous protein with CHL1 in *Vitis vinifera* and named it VvNPF6.5 (Fig. S1). The symbol numbering used follows that suggested by Léran et al., 2014 [20]. Full-length sequence of *VvNPF6.5* was amplified from cDNA of ‘Thompson Seedless’ grapevines using primers listed in Table S1. The sequence of *VvNPF6.5* was determined and the deduced protein sequence showed 67% identity with CHL1 and 56% identity with OsNRT1.1B (Fig. 1). Like CHL1 and OsNRT1.1B, VvNPF6.5 was found to contain the major facilitator superfamily (MFS) domain with 12 putative transmembrane domains and a long hydrophilic loop between transmembrane domains 6 and 7 (Fig. 1).

### VvNPF6.5 is localized to the plasma membrane

**Fig. 2:**
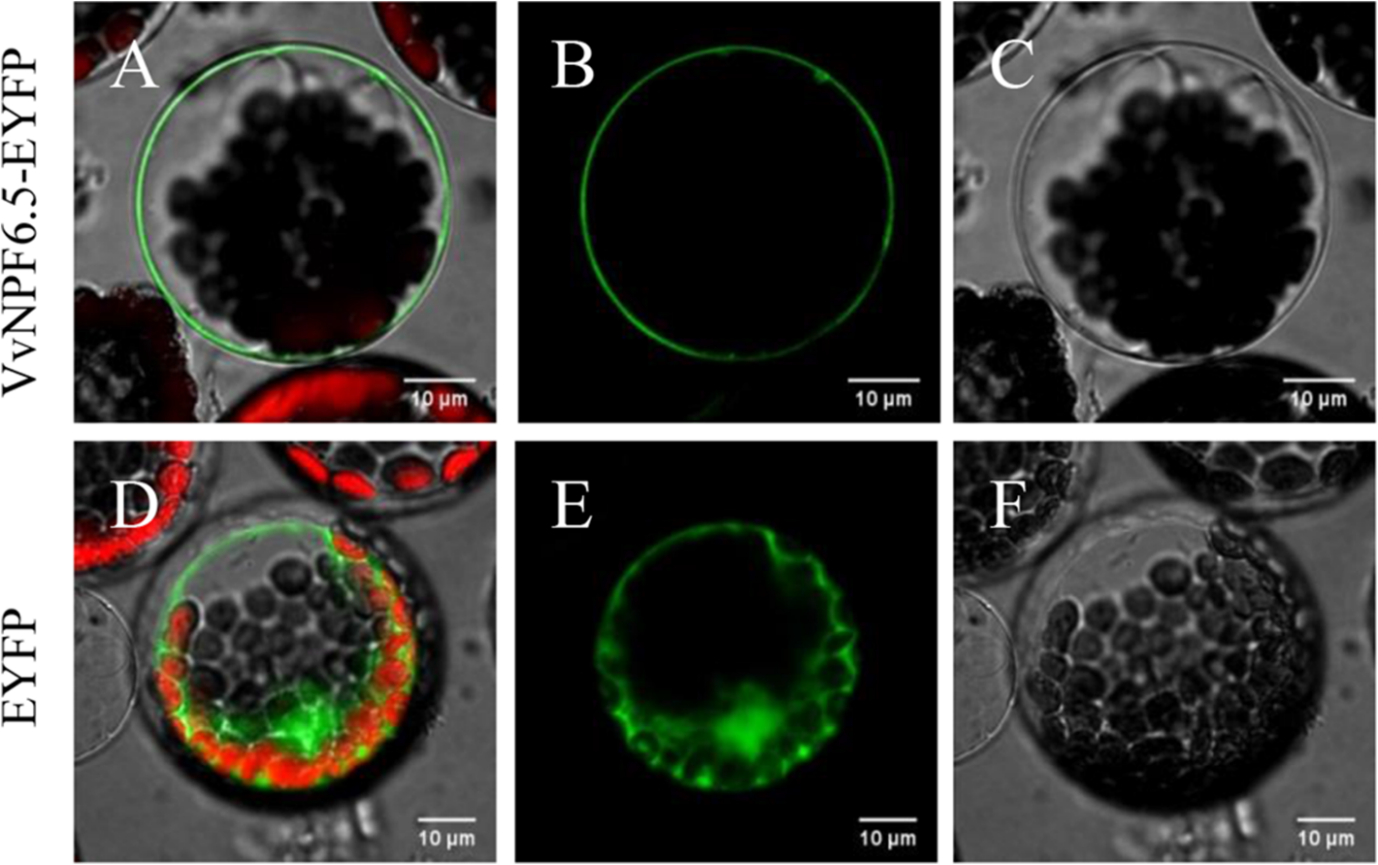
VvNPF6.5 is located in the plasma membrane. *VvNPF6.5-EYFP* fusion construct (**a**-**c**) or unfused *EYFP* (**d**-**f**) was transiently expressed in Arabidopsis mesophyll protoplasts. **a** and **d**, merged fluorescence images of EYFP and chlorophyll. **b** and **e**, EYFP fluorescence only. **c** and **f**, bright-field images. Red color represents autofluorescence of chlorophyll. Bars = 10 μm

To investigate the subcellular localization of VvNPF6.5, VvNPF6.5 was fused in frame with the EYFP under the control of CaMV 35S and the fusion protein was transiently expressed in Arabidopsis mesophyll protoplasts. The fluorescence was seen in cytoplasm in the EYFP control, while the fluorescence signal of VvNPF6.5-EYFP formed a ring outside the chloroplast signal, indicating that VvNPF6.5 is localized in the plasma membrane (Fig. 2).

### VvNPF6.5 is a pH-dependent dual-affinity nitrate transporter

**Fig. 3:**
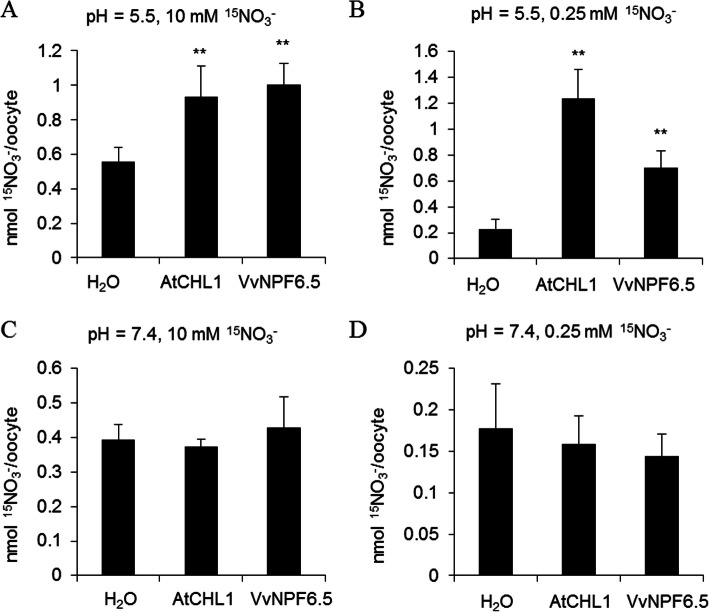
VvNPF6.5 is a pH dependent dual-affinity nitrate transporter. **a** and **b**, Nitrate uptake activity of *VvNPF6.5*-, *AtCHL1*-, or H_2_O-injected oocytes at pH 5.5. Oocytes were incubated with 10 mM ^15^NO_3_^−^ (**a**) or 0.25 mM ^15^NO_3_^−^ (**b**) at pH 5.5 for 3 h. ^15^N retained in the oocytes was measured as described in methods. Values are means± SD (*n* = 5–10). **c** and **d**, Nitrate uptake activity of *VvNPF6.5*-, *AtCHL1*-, or H_2_O-injected oocytes at pH 7.4. Oocytes were incubated with 10 mM ^15^NO_3_^−^ (**c**) or 0.25 mM ^15^NO_3_^−^ (**d**) at pH 7.4 for 3 h. Values are means± SD (*n* = 5–7). Asterisks indicate difference at *P* < 0.01 (**) compared with the H_2_O-injected oocytes by Student’s *t*-test

To elucidate the function of VvNPF6.5, it was heterologously expressed in *Xenopus laevis* oocytes and the nitrate uptake activity was assessed by analyzing ^15^NO_3_^−^ uptake activity. Because the *K*m values of plants with an HATS are in the micromolar range, whereas the LATS exhibits linear kinetics or *K*m values in the millimolar range [11], 10 mM or 0.25 mM nitrate were used to determine the nitrate uptake activity, as previously reported [13, 21–23]. Compared with water-injected oocytes, VvNPF6.5-injected oocytes showed enhanced ^15^NO_3_^−^ uptake activity when incubated with 10 mM ^15^NO_3_^−^ or 0.25 mM ^15^NO_3_^−^ at pH 5.5 (Fig. 3a, b), consistent with the positive control CHL1, indicating that VvNPF6.5 is a nitrate transporter with both the low-affinity and the high-affinity uptake activity. However, the ^15^NO_3_^−^ uptake activity of VvNPF6.5-injected oocytes at pH 7.4 was considerably lower than that at pH 5.5 (Fig. 3c, d), and comparable with water-injected oocytes, indicating that VvNPF6.5 was a proton-coupled nitrate transporter. Taken together, these findings indicate that VvNPF6.5 functions as a pH-dependent dual-affinity nitrate transporter.

### Expression pattern of *VvNPF6.5*

**Fig. 4:**
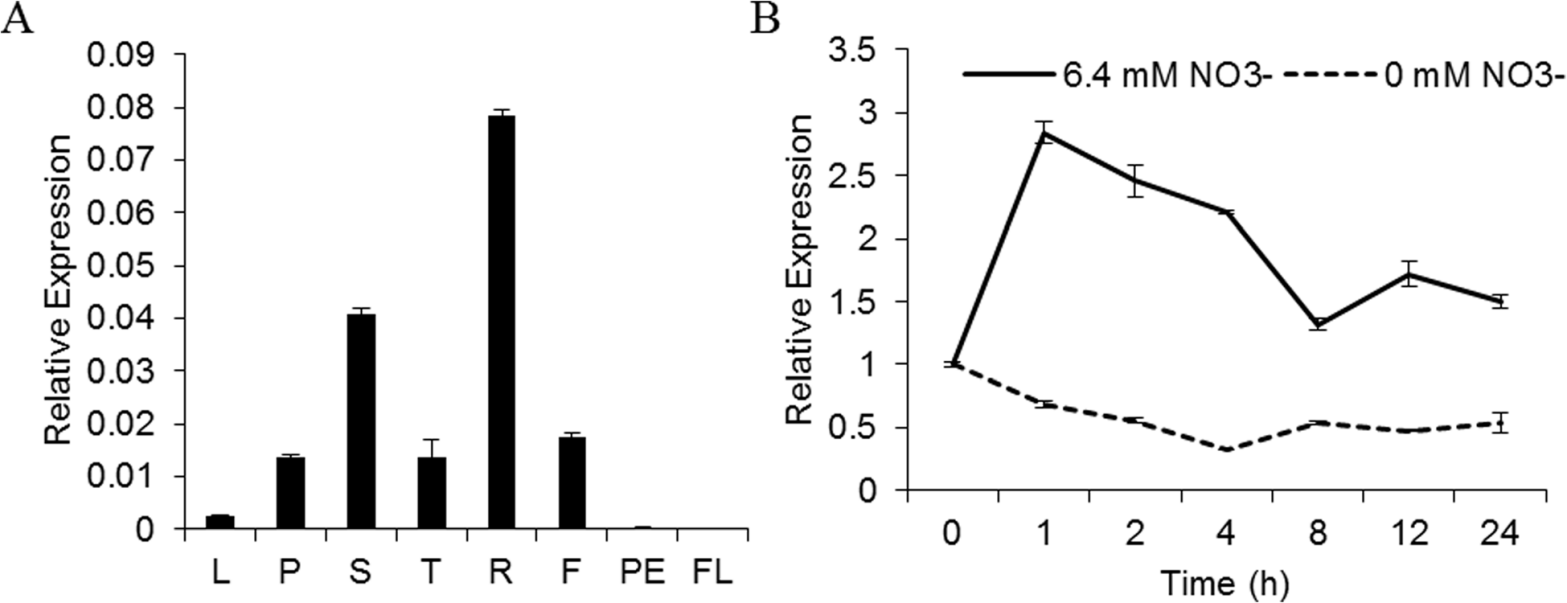
Expression pattern of *VvNPF6.5*. **a**, Tissue-specific expression of *VvNPF6.5.* qRT-PCR analysis was used to determine the relative expression level of *VvNPF6.5* in various tissues and organs. L, leaf blades; P, petioles; S, stems; T, tendrils; R, roots; F, flowers; PE, peel; FL, flesh. Relative expression of *VvNPF6.5* was normalized to that of *VvACT1*. **b**, Time-course analysis of *VvNPF6.5* expression following nitrate induction. ‘Kyoho’ seedlings were grown in soil for 2 months and then grown hydroponically in nitrogen-starvation medium for 1 week. The plants were subjected to 6.4 mM NO_3_^−^ or 0 mM NO_3_^−^ for the indicated time. Total RNA was isolated from root tips and *VvACT1* was used as the internal control. The relative level is the expression normalized to that of 0 h. Values are means ± SD, *n* = 3

The tissue specific expression of genes could provide hint for the physiological function of the coding proteins. To explore the expression pattern of *VvNPF6.5*, qRT-PCR was performed in different grapevine tissues. As shown in Fig. 4a, the expression level of *VvNPF6.5* was higher in roots and stems than that in petioles, tendrils and flowers, and was hardly detected in leaves, peel and flesh. To investigate whether *VvNPF6.5* was nitrate-inducible, grapevine seedlings were shifted from nitrogen starvation medium to nitrate medium and root tissues were harvested to analyze. As shown in Fig. 4b, *VvNPF6.5* expression was rapidly induced within 1 h (more than 4-fold) and declined gradually thereafter, but it was still higher than that without nitrate for at least 24 h. Those expression analyses of *VvNPF6.5* imply that it might be involved in nitrate absorption.

### Ectopic expression of VvNPF6.5 improved nitrate content and NUE in Arabidopsis

**Fig. 5:**
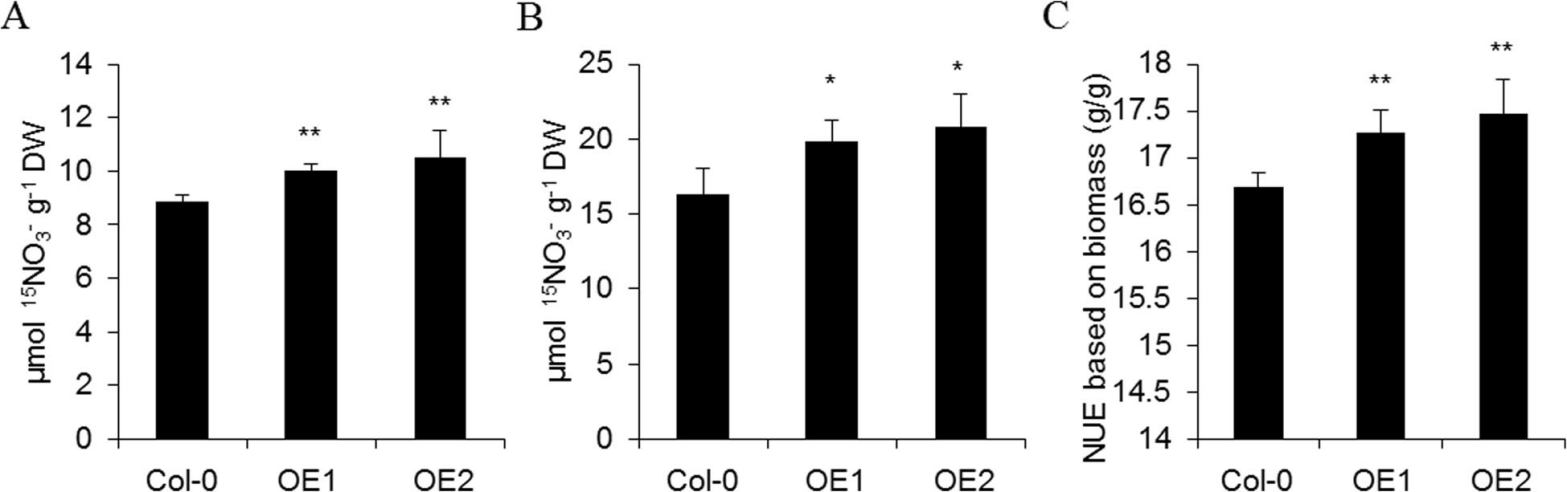
Overexpression of *VvNPF6.5* enhances nitrate accumulation and NUE in Arabidopsis. **a** and **b**, Plants were grown hydroponically for 24 days and treated with 2.25 mM K^15^NO_3_ for 30 min. ^15^N contents in shoots (**a**) and roots (**b**) were analyzed. **c**, Plants were grown hydroponically for 24 days and shoots and roots were harvested to analyze the dry biomass and total N. Values are means ± SD, *n* = 5. Asterisks indicate difference between wild type and overexpression lines at *P* < 0.05 (*) and *P* < 0.01 (**) by Student’s *t*-test

To investigate VvNPF6.5 function, VvNPF6.5 was transformed into wild-type Arabidopsis (Col-0) under the control of 35S promoter and two representative lines (#9 and #20) *OE1* and *OE2* were chosen for further phenotypic analysis (Fig. S2). When *VvNPF6.5* ectopic overexpression lines were fed with ^15^NO_3_^−^ for 30 min, ^15^N concentrations in shoots of *OE1* and *OE2* were increased 13.23 and 18.63%, respectively, and ^15^N concentrations in roots of *OE1* and *OE2* were increased 21.54 and 27.90%, respectively, compared with that of wild-type plants (Fig. 5a, b). However, there is no difference of nitrate concentration between the wild type control and the transgenic plants (Fig. S3). The observation was reasonable because the excess uptake nitrate could further participate in nitrate assimilation. In addition, we checked NUE of the Arabidopsis transformants by determining the dry weight and total nitrogen content [24]. As shown in Fig. 5c, the NUE of *OE1* and *OE2* was improved 4.48 and 5.89% compared with that of wild type. These results suggested that VvNPF6.5 is involved in nitrate uptake from soil and root-to-shoot transport and further influence the NUE.

### VvNPF6.5 is involved in the primary nitrate response

**Fig. 6:**
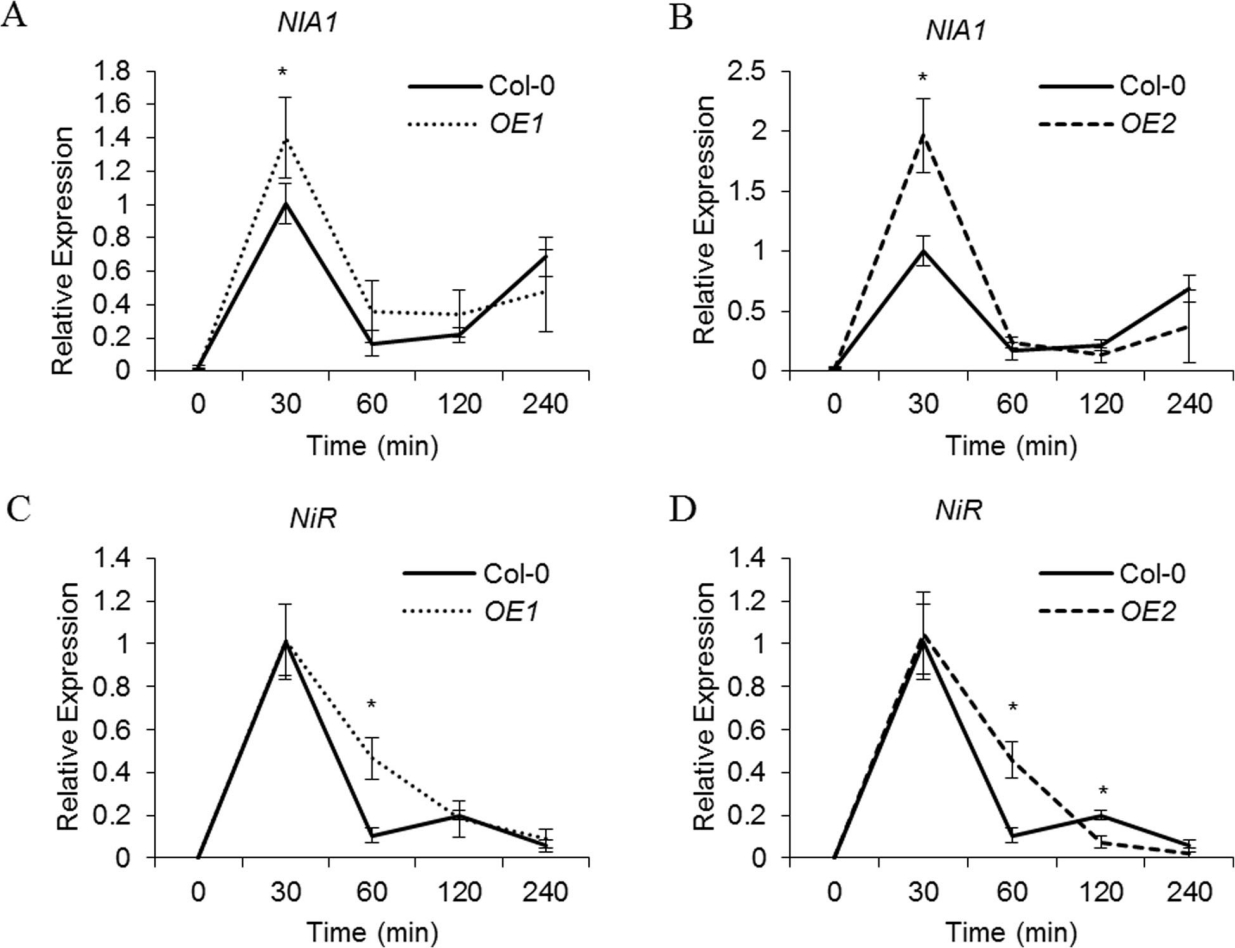
Induction levels of nitrate-regulated genes are improved in *VvNPF6.5* transgenic plants. The expression of *NIA1* (**a** and **b**) and *NiR* (**c** and **d**) was analyzed by quantitative RT-PCR. Wild-type and overexpression lines were grown hydroponically for 24 days, after which they were treated with nitrate-free medium using NH_4_HCO_3_ as the nitrogen source for 4 days and then exposed to 10 mM NO_3_^−^ for the indicated time. The relative expression level was calculated by normalizing to that in wild-type plants which exposed to 10 mM NO_3_^−^ for 30 min. *AtUBQ10* was used as the internal control. Values are means ± SD, *n* = 3. Asterisks indicate difference between wild type and overexpression lines at *P* < 0.05 (*) by Student’s *t*-test

As an important signal, nitrate can rapidly induce the expression of several nitrate-related genes [12]. The rapid transcriptional response has been referred to as the primary nitrate response [25]. Expression of *VvNPF6.5* was rapidly induced by nitrate as showed in Fig. 4b. To determine whether VvNPF6.5 is involved in the primary nitrate response, the expression of two nitrate-induced genes, the nitrate reductase 1 (*AtNIA1*) and the nitrite reductase gene (*AtNiR*), was analyzed in *VvNPF6.5* overexpression lines [26]. When wild-type plants were exposed to 10 mM nitrate, *AtNIA1* and *AtNiR* were highly induced within 30 min (Fig. 6), which was consistent with previous studies [26]. The same treatment was carried out in *OE1* and *OE2*, and we found that *AtNIA1* and *AtNiR* were also induced by nitrate with a similar time course, while the induction levels were significantly higher than those in the wild type (Fig. 6). Collectively, these data suggested that VvNPF6.5 might also participate in the primary nitrate response.

## Discussion

Nitrate transporters have been reported in Arabidopsis, rice, wheat, tomato and other species [10, 27–29]. However, only VvNPF3.2 was identified in grapevines, which functions as a low-affinity transporter for both nitrate and nitrite and plays a role in host nutrient distribution during powdery mildew infection [30]. In this study, we characterized another nitrate transporter, VvNPF6.5, as a dual-affinity nitrate transporter mainly expressed in roots and stems, and demonstrated that VvNPF6.5 is responsible for nitrate uptake and allocation in grapevines.

*VvNPF6.5* belongs to the family of NRT1/PTR/NPF. In this family, only CHL1 and its homology proteins MtNRT1.3 and OsNRT1.1B behave as dual-affinity nitrate transporters, while other members are all characterized as low-affinity nitrate transporters [31, 32]. The high- and low-affinity nitrate uptake activities of VvNPF6.5 were assessed by analyzing ^15^NO_3_^−^ uptake activity of cRNA-injected Xenopus oocytes at 10 mM and 0.25 mM (Fig. 3). Interestingly, VvNPF6.5 also functions as a dual-affinity nitrate transporter. Sequence alignment revealed that RXXT^100^ in *VvNPF6.5* is identical to the phosphorylation site RXXT^101^ in *CHL1*, which is responsible for the shift between low- and high-affinity (Fig. 1). The regulatory mechanism allows plants to change the affinity of nitrate uptake rapidly, which might be critical when competing for limited nitrate [14]. However, the site is also found in other low affinity nitrate transporters, indicating there might be additional regulatory mechanisms.

The expression of *VvNPF6.5* was induced by nitrate (Fig. 4b), similar to those nitrate transporter genes which regulate nitrate uptake, like *CHL1*, *AtNRT2.1* and *AtNRT2.1* [11]. The strong correlation between the transcript abundance of these nitrate transporter genes and nitrate uptake activities suggests that transcriptional regulation plays important role in modulating nitrate uptake activities [11]. In addition to its absorption properties, VvNPF6.5 was found to participate in nitrate long-distance transport because the expression level of *VvNPF6.5* was relatively high in stems and the ^15^N content in shoots of overexpression lines was also increased after ^15^NO_3_^−^ feeding (Fig. 5a). It is of great significance to identify the transporter involved in nitrate long-distance transport because the process determines nitrate distribution and subsequent assimilation between different tissues and further influence the nutrient use efficiency [33].

Several nitrate transporters have been reported to contribute to nitrogen use efficiency. OsNRT1.1B is an important player in NUE improvement for crop breeding because its polymorphism could result in NUE difference [31]. Coordinate regulation of *BnNRT1.5* and *BnNRT1.8* could promote nitrate allocation to aerial parts and contribute to higher NUE in *Brassica napus* [24]. Increasing the expression of *OsNRT2.1*, especially under the control of *OsNAR2.1* promoter, can improve the yield and NUE in rice [34]. In this study, VvNPF6.5 was proved to play role in regulating NUE in grapevines because transgenic plants showed higher NUE compared to wild type in Arabidopsis (Fig. 5c). These studies showed that nitrate transporters could improve NUE by regulating nitrate uptake or long-distance translocate, indicating that nitrate acquisition was very important and providing a new perspective for the study of NUE.

Nitrate functions as not only a nutrient but also a signaling molecule. Nitrate signaling participates in regulating many physiological processes like root development, shoot growth, ABA-independent stomatal opening, flowering time and seed dormancy [17, 35]. The primary nitrate response is the best known nitrate-induced response, in which the expression of several nitrate transporters and nitrate assimilatory enzymes is rapidly induced under nitrate treatment [36]. Time-course analysis of *VvNPF6.5* expression showed that *VvNPF6.5* was rapidly and highly induced by nitrate (Fig. 4b), which was consistent with the primary nitrate response. Therefore, the effect of VvNPF6.5 on nitrate signaling was further detected. In *VvNPF6.5* overexpression lines, the rapid nitrate-induced expression of the two known primary nitrate response genes, *NIA1* and *NiR*, was enhanced (Fig. 6), suggesting that VvNPF6.5 might be involved in nitrate signaling. CHL1 was proved to function as a nitrate sensor for nitrate signaling in the primary nitrate response [16]. It has been speculated that such a function may not be restricted to CHL1 and may concern other NRT1/PTR/NPF proteins from different species [37]. VvNPF6.5 is an interesting candidate in grapevines, while further research is required to test whether VvNPF6.5 is also a nitrate sensor.

Given that the ^15^NO_3_^−^ content in roots and shoots was increased (Fig. 5a, b) and the primary nitrate response was enhanced when *VvNPF6.5* was overexpressed (Fig. 6), it is a possibility that grapevines roots utilize a transporter responsible for nitrate uptake and translocate, such as VvNPF6.5, to monitor changes of nitrate concentrations in soil and in turn this could induce the expression of nitrate-related genes and then regulate nitrogen metabolism. Therefore, the results indicate that the higher NUE of overexpression lines might be achieved by regulating nitrate uptake, root-to-shoot nitrate long-distance transport and nitrate signaling.

## Conclusions

In this study, we identified and functional characterized a nitrate transporter, VvNPF6.5, in grapevines. VvNPF6.5 was localized to plasma membrane and functioned as a pH-dependent, dual-affinity nitrate transporter. Overexpression of *VvNPF6.5* in Arabidopsis could increase nitrate content in shoots and roots and improve the NUE, indicating that VvNPF6.5 regulates nitrate absorption and allocation. Additionally, *VvNPF6.5* was proved to play important role in the primary nitrate response in grapevines. This study would offer a foundation for further exploration into nitrogen use in grapevines and provide a candidate gene for the improvement of NUE in grapevines. In addition, this study would also provide a method reference for the identification of other nitrate transporters.

## Methods

### Plant materials and growth conditions

The Arabidopsis (*Arabidopsis thaliana*) ecotype Columbia-0 (Col-0) was acquired from the Arabidopsis Biological Resource Center (https://abrc.osu.edu/) and was used as the wild-type control. *OE1* and *OE2* were two T3 homozygous lines generated by transforming *VvNPF6.5* into Col-0 under the control of cauliflower mosaic virus 35S (CaMV 35S) promoter (Fig. S4), using the floral dip method [38]. The wild-type Col-0, rather than the homolog gene mutant *chl1* was used for transformation because there was no direct evidence to show that the nitrate content in *chl1* was lower. The wild type and overexpression lines were grown in hydroponic solution at 22 °C with 16-h-light/8-h-dark cycles as described [39]. At 3 to 4 weeks of age, plants were exposed to treatments as indicated in the legends of Figure.

Grapevines were obtained from Shenyang Changqing grape Technology Co., Ltd. 10-year-old ‘Muscat Kyoho’ (*V. vinifera* × *V. labrusca*) and ‘Kyoho’ (*V. vinifera* × *V. labrusca*) seedlings were cultivated in soil or soilless growth systems in the greenhouse located in the Shanghai Academy of Agricultural Sciences, Shanghai, China (30°51’N, 121°13’E). Soilless growth was carried out in 5 L pots containing modified Hoagland’s nutrient solution (in mM: NO_3_^−^, 6.40; PO_4_^3−^, 0.50; K^+^, 2.50; Ca^2+^, 2.20; Mg^2+^, 1.20; SO_4_^2−^, 1.20; and in μM: Fe^2+^, 30.00; Na^+^, 30.00; Mn^2+^, 3.50; NH_4_^+^, 0.30; BO_3_^3−^, 17.50; Zn^2+^, 0.25; MoO_4_^2−^, 0.05; Cu^2+^, 0.125). Nitrogen-starved nutrient solution was made up by replacing KNO_3_ and Ca (NO_3_)_2_ with KCl and CaCl_2_. Random sampling of different organs was performed from plants grown in the same plot. Leaf blades, petioles, stems, tendrils and roots were sampled at the vegetative development stage. Flowers were sampled at full bloom and peel and flesh were sampled at mature stage.

### Cloning and sequence analysis of VvNPF6.5

VvNPF6.5 was the best matched protein when using CHL1 as the query sequence by protein-protein BLAST in *V. vinifera* (https://blast.ncbi.nlm.nih.gov/Blast.cgi). Phylogenetic tree of all members of NRT1/PTR/NPF transporters in *Arabidopsis thaliana* (https://www.arabidopsis.org/) and *Vitis vinifera* (http://plants.ensembl.org/index.html) was constructed and VvNPF6.5 shared the highest degree of sequence similarity with CHL1 [20]. *VvNPF6.5* was thus selected and was amplified by PCR using the primers listed in Table S1. The PCR product was cloned into the pGEM-T easy vector (Promega, USA) and was then sequenced. NCBI-Conserved Domain Data (CDD) search were used to identify the conserved domain (https://www.ncbi.nlm.nih.gov/Structure/cdd/wrpsb.cgi). The transmembrane region was determined according to Sun et al., 2014 [40].

### Functional analysis of VvNPF6.5 in *Xenopus laevis* oocytes

The 1.8-kb *VvNPF6.5* cDNA was cloned into the oocyte expression vector pOO2 [41]. cRNA was synthesized as described previously [19]. Oocytes from multiple frogs were isolated and injected with 50 ng of *VvNPF6.5* cRNA in 50 nL of water, as described previously [12], except that the Barth solution was replaced with ND96 (96 mM NaCl, 2 mM KCl, 1 mM MgCl_2_, 1.8 mM CaCl_2_, and 5 mM HEPES, pH 7.4) [42]. *CHL1* cRNA- and water- injected oocytes were used as positive and negative controls, respectively. At least 30 good quality oocytes per gene were injected for each treatment. The oocytes were incubated for 2 d in ND96 solution, after which the ones that are injured, misshapen, or off-color were discarded and the large, evenly rounded oocytes with a creamy half and a tan or brown half with a clear division line or equatorial band were selected for further experiments [43]. The frogs used would recover consciousness after the experiment and would be placed back to its tank. A frog could provide oocytes up to four times, with an interval of at least 3 months [43]. After that, the frogs would continue to be kept for several years until they dead naturally, and their bodies would be handed over to the animal facility for further processing. For the analysis of uptake properties by ^15^N measurements, oocytes were incubated for 3 h in a solution containing 230 mM mannitol, 0.3 mM CaCl_2_, 10 mM MES-Tris, pH 5.5 or 7.4, and the required concentration of K^15^NO_3_ and then were rinsed six times with ND96 buffer and dried at 80 °C for 48 h [23]. The retained ^15^N of each oocyte was then measured on a continuous-flow isotope ratio mass spectrometer coupled to a carbon nitrogen elemental analyzer (Thermo Fisher Scientific DELTA V Advantage).

### Subcellular localization by Arabidopsis protoplast transformation

*VvNPF6.5* cDNA was amplified using the primers listed in Table S1 and the amplified DNA fragment was cloned in frame in front of EYFP in the vector 35S::*EYFP*/PA7, leading to the final VvNPF6.5-EYFP construct under the control of the CaMV 35S promoter. The fusion construct or the vector 35S::*EYFP*/PA7 was then transiently expressed in Arabidopsis protoplasts using the method described before [44]. Arabidopsis protoplasts were isolated from leaf tissues of 3- to 4-week-old plants grown on soil. *EYFP*-fused plasmids isolated by QIAGEN Plasmid Kits (QIAGEN, Germany) were transformed into protoplasts. After incubation in W5 solution in dark for more than 24 h, fluorescent cells were imaged using confocal microscopy (Nikon C2-ER).

### RT-PCR and quantitative RT-PCR

RNA was extracted using RNAprep Pure Plant Plus Kit (TIANGEN, China) according to the manufacturer’s instructions. First-strand cDNA was synthesized using Takara PrimeScript™ RT reagent Kit with gDNA Eraser (Takara, Japan). PCR was performed using Hieff™ PCR Master Mix (Yeasen, China). Quantitative RT-PCR was performed on a LightCycler 480 System (Roche, Germany) using SYBR Premix Ex-Taq (TaKaRa, Japan) according to the manufacturer’s protocols. Primers used in the assays are listed in Table S1.

### Analysis of NUE

Plants were harvested and dried in an oven for 30 min at 105 °C, then at 80 °C to a constant weight and weighed. N concentration was determined using a carbon nitrogen elemental analyzer (Thermo Fisher Scientific DELTA V Advantage). NUE values based on dry biomass were calculated as the proportion of biomass/total N in plants [24].

### ^15^NO_3_^−^ labeling assay

Wild type and overexpression lines were grown hydroponically for 24 days old and then were treated with hydroponics medium containing 2.25 mM K^15^NO_3_ with 99% atom excess of ^15^N for 30 min. The shoots and roots were separated and harvested, and the ^15^N content was detected as described [19].

### Statistical analysis

Two-tailed Student’s *t*-tests were performed. Differences were deemed significant (*) at *P* < 0.05 and extremely significant (**) at *P* < 0.01.

## Supplementary Information

**Additional file 1.**

## Data Availability

Sequence of VvNPF6.5 was deposited in GenBank and the accession number is MW094160.

## Abbreviations

NRT1/PTR/NPF: Nitrate transporter 1/Peptide transporter family
NUE: Nitrogen use efficiency
HATS: High affinity transport system
LATS: Low-affinity transport system
NRT2: Nitrate transporter 2 family
CLC: Chloride channel family
SLAC/SLAH: Slow anion channel-associated homologues
CaMV 35S: Cauliflower mosaic virus 35S promoter
*AtNIA1*: Nitrate reductase 1
*AtNiR*: Nitrite reductase gene
