# Supplementary material for: Ectopic expression of a grape nitrate transporter VvNPF6.5 improves nitrate content and nitrogen use efficiency in Arabidopsis
Source: BMC Plant Biol. 2020 Dec 7;20:549. doi: 10.1186/s12870-020-02766-w (PMC7722303; doi:10.1186/s12870-020-02766-w)
Supplement: Supplementary file 1 — Additional file 1. [file 12870_2020_2766_MOESM1_ESM.pdf]

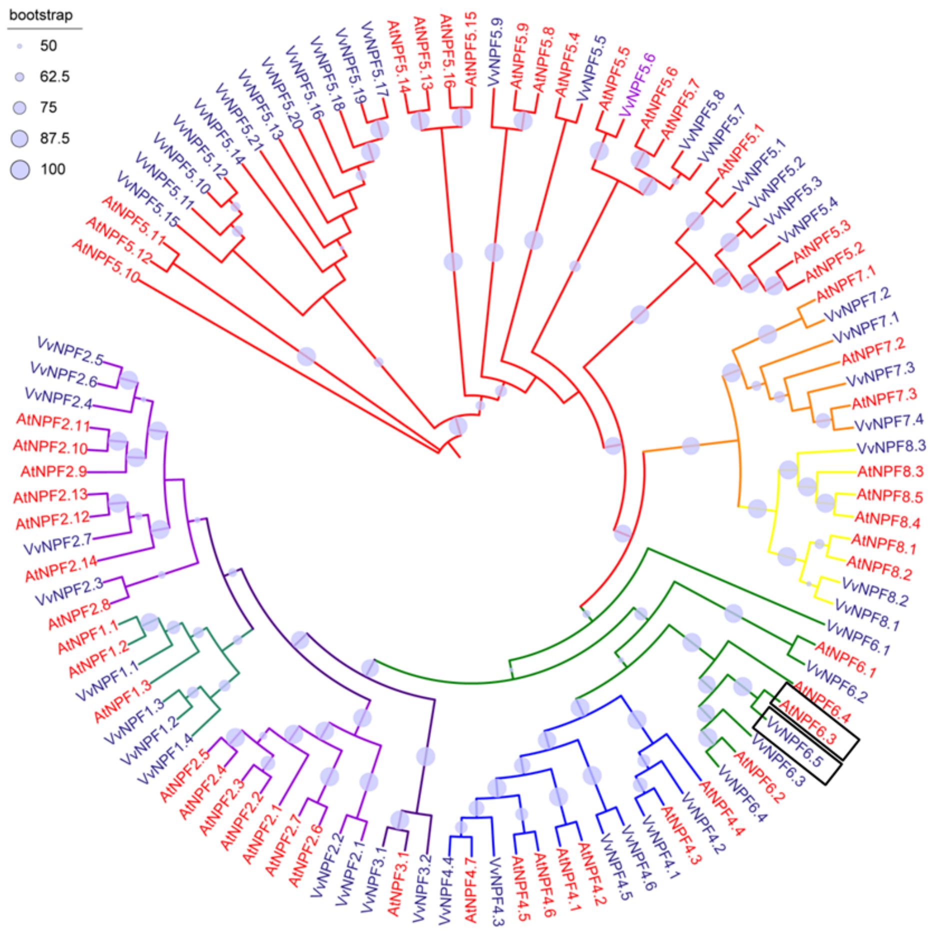

Figure S1. Phylogenetic tree of NRT1/PTR/NPF family in *Arabidopsis thaliana* and *Vitis vinifera*. The tree was produced using IQ-Tree with the LG+F+R6 model. Phylogenetic support was estimated using 1,000 ultrafast bootstrap replicates. Bootstrap values are displayed on branches. AtNPF6.3 (CHL1) and VvNPF6.5 are enclosed in the black box.

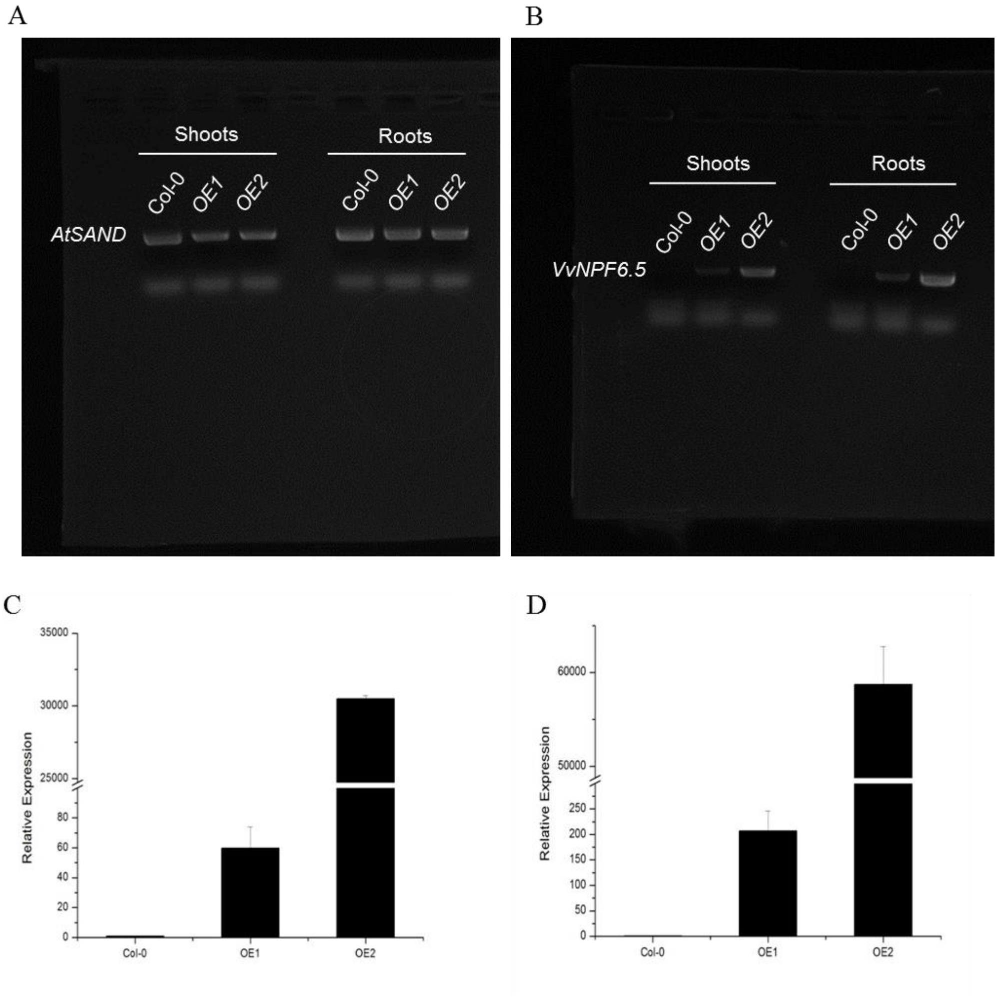

Figure S2. Identification of *VvNPF6.5* overexpression lines. *VvNPF6.5* overexpression lines were identified by RT-PCR (A, B) and qRT-PCR(C, D). *Arabidopsis* were grown hydroponically for 24 days and the shoots (A, C) and roots (B, D) were sampled and analyzed. A and B, *AtSAND* was used as a loading control which was amplified for 28 cycles, *VvNPF6.5* was amplified for 32 cycles in Col-0 and *OE1* and 23 cycles in *OE2*. C and D, *AtSAND* was used as the internal control. Data were normalized to that of wild type. Values are means ± SD, n = 3.

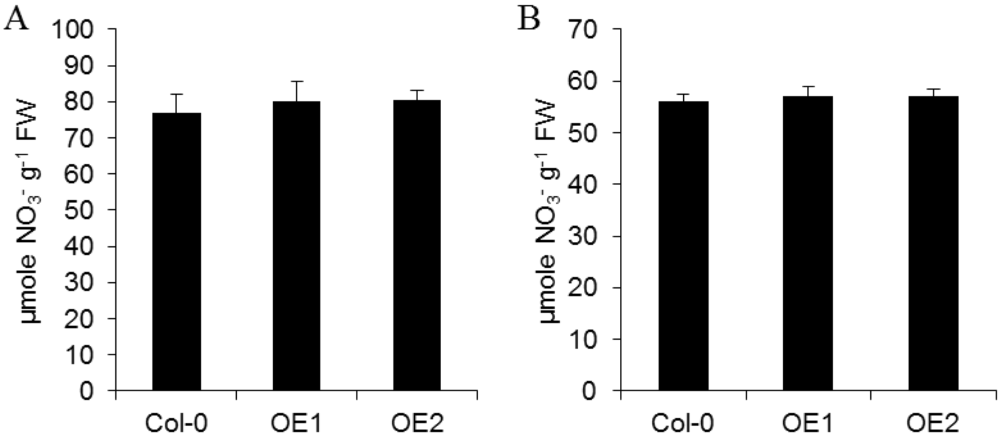

Figure S3. Overexpression of *VvNPF6.5* does not affect nitrate accumulation in shoots and roots. Plants were cultivated in hydroponics for 24 d, then the shoots (A) and roots (B) of wild-type plants and overexpression lines were harvested to analyze nitrate concentration. Values are means ± SD, n = 3.

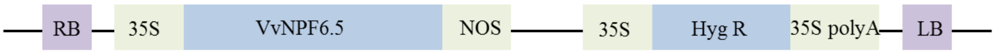

Figure S4. Structure of the CaMV 35S promoter- VvNPF6.5 ectopic expression construct. LB, left border; RB, right border; 35S, CaMV 35S promoter; NOS, terminator; Hyg R, hygromycin resistance.

Table S1. List of primer sequences

AtNIA1-qRT-F	ACTAGGGCACATCGAG
AtNIA1-qRT-R	TGCTTACTAGCCCATCC
AtNiR-qRT-F	GTGGCTTATCGACGAAC
AtNiR-qRT-R	CCTGACCCGTAGGTAT
AtSAND-qRT-F	ATATGACACCCTTGCTTGGAGGGA
AtSAND-qRT-R	TGAGAATAAGACACCAGACGCGCA
AtUBQ10-qRT-F	CTTCGTCAAGACTTTGACCG
AtUBQ10-qRT-R	CTTCTTAAGCATAACAGAGACGAG
VvACT1-qPCR-F	CTTGCATCCCTCAGCACCTT
VvACT1-qPCR-R	TCCTGTGGACAATGGATGGA
VvNPF6.5-qPCR-F	ATCCCACCTGCATCACTAAC
VvNPF6.5-qPCR-R	AAGTATCCTGGCTGCTATTGG
VvNPF6.5-F	GCCTCTTCAACCAATTCAGAC
VvNPF6.5-pA7-R	GTGACAAGTAGGGCCAACGT
VvNPF6.5-pOO2-R	TTAGTGACAAGTAGGGCCAACGT
